# Phase separation-mediated biomolecular condensates and their relationship to tumor

**DOI:** 10.1186/s12964-024-01518-9

**Published:** 2024-02-21

**Authors:** Xi Wang, Jiameng Liu, Chaoming Mao, Yufei Mao

**Affiliations:** 1 Department of Nuclear Medicine, The Affiliated Hospital of Jiangsu University, Zhenjiang, 212001 China; 2 Department of Ultrasound Medicine, The Affiliated Hospital of Jiangsu University, Zhenjiang, 212001 China

**Keywords:** Phase separation, Biomolecular condensates, Tumor, Therapeutic targets

## Abstract

Phase separation is a cellular phenomenon where macromolecules aggregate or segregate, giving rise to biomolecular condensates resembling "droplets" and forming distinct, membrane-free compartments. This process is pervasive in biological cells, contributing to various essential cellular functions. However, when phase separation goes awry, leading to abnormal molecular aggregation, it can become a driving factor in the development of diseases, including tumor. Recent investigations have unveiled the intricate connection between dysregulated phase separation and tumor pathogenesis, highlighting its potential as a novel therapeutic target. This article provides an overview of recent phase separation research, with a particular emphasis on its role in tumor, its therapeutic implications, and outlines avenues for further exploration in this intriguing field.

## Introduction

Cells have evolved intricate membrane systems to uphold normal physiological functions, effectively compartmentalizing various activities within distinct spaces and timeframes. These compartments, known as membrane organelles (MBO) or classical organelles, include familiar structures like mitochondria, the endoplasmic reticulum (ER), and the Golgi apparatus. Beyond these membrane-bound organelles, cells also harbor membraneless organelles (MLO), intriguing structures devoid of the typical lipid membrane but instead formed through multivalent interactions among biomolecules, creating membraneless compartments. Examples of MLO encompass the nuclear kernel, P granules, stress granules (SGs), Promyelocytic Leukemia (PML) bodies, and nuclear granules [1]. Termed biomolecular condensates, MLO result from the spontaneous polymerization of biomolecules via liquid–liquid phase separation (LLPS). Comprising primarily proteins and nucleic acids, MLO reside within the nucleus or cytoplasm, possessing liquid-like fluidity, enabling free flow, deformation, fission akin to droplets, and active interactions with the surrounding liquid milieu [2], which is the basis for maintaining a variety of physiological functions [3–7].

However, when phase separation occurs inappropriately regarding timing, location, or environment, it can lead to abnormal biomolecular condensation, heightening the risk of tumor [8]; Conversely, if the liquid condensate transitions into a solid aggregate and impedes molecular exchange with the surrounding environment, it can predispose individuals to neurodegenerative diseases such as Alzheimer's disease (AD) [9]. Studies have elucidated that these abnormal phase separations may impact critical processes such as DNA repair, transcriptional regulation, the expression of proto-oncogenes and tumor suppressor genes, and the formation of PML bodies and SGs [10]. This article predominantly explores the intricate link between LLPS and tumor at the molecular level, offering insights into the physiological functions, driving forces, and regulatory mechanisms of phase separation. Additionally, it delves into the mechanisms and therapeutic prospects of biomolecular condensates arising from anomalous phase separation in the context of tumor, aiming to provide innovative avenues for tumor treatment.

### Phase separation overview

In the realm of cell biology, phase separation refers to the physiological process wherein a single phase comprising diverse components spontaneously segregates into two or more coexisting phases within cells [11], a phenomenon also recognized as LLPS. Proteins and nucleic acids within cells become segregated from their surrounding environment through intramolecular or intermolecular interactions, culminating in the formation of autonomous phases termed MLO or biomolecular condensates. These biomolecular condensates exhibit molecular compositions akin to their surroundings but diverge in concentration. When their concentration surpasses solubility thresholds, they undergo condensation and separation from the solution, resembling the formation of droplets. The attributes of phase separation predominantly involve the creation of biomolecular condensates that are spherical, undergo dynamic fusion, and exhibit droplet recovery after photobleaching [12] and the proteins undergoing phase separation often possess characteristic sequence features, such as intrinsically disordered regions (IDRs) and multiple modular binding domains, within which multivalent weak interactions drive the process [13]. These biomolecular condensates have the potential to regulate a myriad of enigmatic biological processes.

In recent years, the field of phase separation has emerged as a prominent research topic within the life sciences. A growing body of evidence underscores the pivotal role of phase separation-mediated biomolecular condensates in cellular functions and their intimate association with a diverse array of diseases [14]. The exploration of membraneless organelles dates back to the 1830s when the first such organelle, the nucleolus, was identified within neuronal nuclei [15]. Subsequently, Wilson et al. in 1899 elucidated the presence of membraneless organelles, including nucleoli, within cells, formed through the condensation of various substances, and attributed specific physiological functions to them [16]. During the 1970s and 1980s, extensive research emerged, delving into the molecular mechanisms that govern the development of intricate condensates within cells [17, 18]. Brangwynne and Hyman in 2009 observed the spontaneous condensation of P granules into droplet-like structures in Caenorhabditis elegans embryos. These droplets exhibited properties akin to liquids, capable of dissolution and concentration while undergoing phase transitions. Photobleaching-recovery experiments revealed their fluidity and dynamic exchange with the surrounding environment, hinting at the role of phase separation in cytoplasmic organization [19]. Further insights came in 2011 when Brangwynne and colleagues discovered that the nucleolus in African clawed frog oocytes comprised RNA and proteins with water droplet-like characteristics, possessing discernible viscous fluid properties [20]. Subsequent research by Li et al. in 2012 demonstrated that various synthetic multivalent macromolecules, encompassing multi-domain proteins and RNA molecules, could undergo phase separation through intermolecular interactions, resulting in the formation of micron-sized droplets in aqueous solutions [21]. Kato and collaborators reconstructed RNA and protein droplets in vitro, mirroring biomolecular condensates in vivo, thereby facilitating the study of these condensates by simulating the in vivo phase separation environment [22]. Lin et al. in 2015 postulated that interactions between RNA and protein-disordered regions could drive the assembly of ribonucleoprotein (RNP) particles via phase separation. This process entailed the transition of RNP particles from a dynamic liquid state to a solid state, subject to intricate regulatory mechanisms, with improper regulation potentially leading to aberrant RNA-related disorders [23]. Building on these findings, Wegmann et al. in 2018 uncovered the role of phase separation in explaining tau protein aggregation in neurodegenerative diseases [24]. Furthermore, Rubio et al. in 2019 delved into the interaction between functional scaffolding proteins, non-coding RNA, and genomic loci, culminating in three-dimensional structure-induced phase separation in cancer. This discovery held promise for the development of novel cancer treatment strategies [25]. Subsequent research has accumulated substantial evidence illustrating the close ties between abnormal phase separation-induced biomolecular condensates and various human diseases, thus opening new avenues for the treatment of major human ailments [26]. Ongoing LLPS research is dynamic, uncovering novel functions of phase separation, such as its impact on cell organization, signal transduction, disease mechanisms, and potential therapeutic interventions (Table 1). As technology and methodologies advance, the trajectory of LLPS research holds promise for unveiling exciting discoveries and applications across various scientific domains.

**Table 1:** Driving forces and functions of common biomolecular condensates

Biomolecular condensates	Drive forces	Functions	References
Nucleoli	RNA-proteins	rRNA synthesis and nascent ribosome assembly	Pederson et al. [15] Brangwynne etal. [20]
P granules	RNA-proteins	Embryonic development	Brangwynne et al. [19]
Stress granules	RNA-proteins	Induce tumor	Protter et al. [23] Tian et al. [27]
Tau protein	Protein IDRs	Induce neurodegenerative diseases	Wegmann et al. [24]
Nucleosomes	DNA-proteins	Chromosome assembly	Gibson et al. [28]
HP1α-H3K9me3	Protein IDRs	Heterochromatin constriction	Larson et al. [29]
Swi6-Nucleosomes	Protein IDRs	Nucleosome assembly	Sanulli et al. [30]
NUP98-HOXA9	Protein IDRs	Induce leukemia transformation	Ahn et al. [31]
Centrosomes,Centromeres, and Mitotic spindles	Protein IDRs DNA-proteins	Cell division	Joseph et al. [4]
EB1-Microtubule	Protein IDRs	Chromosome separation	Song et al. [32]
cGAS-dsDNA	DNA-proteins	Signal transduction	pan et al. [33]
TRAF6-TLR4	Protein IDRs	Signal transduction	Li et al. [34]
PPARγ-RXRα-PPRE	DNA-proteins	Regulation of gene expression	Li et al. [35]
Autophagosomes	Protein IDRs	Initiation of autophagy	Fujioka et al. [36]
PGL particles	RNA-proteins	Escape of autophagy	Zheng et al. [37]
abLIM1 protein	Protein IDRs	Microfilament assembly	Yang et al. [38]
PSD	Protein IDRs	Neural synapse formation	Chen et al. [39]
SH3-PRM proteins	Protein modules	Cell signal transduction	Pilong Li [40]
DDX4	Protein IDRs	Regulation of RNA metabolism	Nott et al. [41]
FUS family proteins	RNA-proteins	Induce neurodegenerative diseases	Wang et al. [42] Qamar et al. [43]
NLRP6-dsRNA	RNA-proteins	Activation of the inflammasome	Shen et al. [44]
P bodies	RNA-proteins	Maintenance of RNA stability	Tian et al. [27]
TDP-43	RNA-proteins	Induce neurodegenerative diseases	Lara et al. [45]
P62-ubiquitination	Protein IDRs	Degradation of autophagy	Sun et al. [46]
UBQLN2	Protein IDRs	Ubiquitination-Proteasomal degradation	Dao et al. [47]
α-syn protein	Protein IDRs	Induce neurodegenerative diseases	Marotta et al. [48]
Nephin-NCK-N-WASP	Protein IDRs	Regulation of actin polymerization in renal podocytes	Jones et al. [49]
LAT-Grb2-Sos1	Protein IDRs	Immune signal transduction	Huang et al. [50]
AGOs-miRNAs	RNA-proteins	Ubiquitination-Proteasomal degradation	Gao et al. [51]
FIP200	Protein IDRs	Initiation of autophagy	Zheng et al. [52]
53BP1-P53	Protein IDRs	DNA damage repair	Kilic et al. [53]
SLX4-XPF	Protein IDRs	DNA damage repair	Alghoul et al. [54]
NONO-SFPQ	Protein IDRs	Tumor radioresistance	Udayakumar et al. [55]
EWS-FLI1	Protein IDRs	Induce Ewing sarcoma	Shirnekhi et al. [56]
EML4-ALK	Protein IDRs	Induce lung cancer	Tulpule et al. [57]
CCDC6-RET	Protein IDRs	Induce thyroid cancer	Tulpule et al. [57]
Enhancers and SEs	Protein IDRs RNA-proteins	Regulation of gene expression	Hnisz et al. [58]
YAP/TAZ	Protein IDRs	Regulation of genes	Lu et al. [59]
Laforin-Mst1/2 complex	Protein IDRs	Induce liver cancer	Liu et al. [60]
kb-1980E6.3-IGF2BP1	RNA-proteins	Induce Breast cancer	Zhu et al. [61]
p53 family proteins	Protein IDRs	Induce tumor	Kanapathipillai et al. [62]
PML bodies	Protein IDRs	Inhibit tumor development	Mu et al. [63]
PML-RARα	Protein IDRs	Induce acute promyelocytic leukemia	Wang et al. [64]
AR-MED1	Protein IDRs	Regulate the expression of androgen-dependent genes	Zhang et al. [65]
icFSP1-FSP1	Protein IDRs	Inhibite tumor	Nakamura et al. [66]

### Physiological functions of phase separation

Extensive research has unveiled the ubiquitous presence of phase separation in living organisms, underscoring its indispensable role in a multitude of vital biological processes. This phenomenon profoundly influences chromosome assembly, cell division, signal transduction, gene expression, autophagy, cytoskeleton assembly, and synapse formation. Phase separation operates as a pivotal mechanism for the creation of membraneless compartments within cells, orchestrating the regulation of their specialized physiological functions through the facilitation of compartmentalization. The condensates arising from phase separation serve as the foundational framework for biomolecules to execute specific functions with precision. Perturbations or irregularities within these condensates can disrupt their normal functionality, leading to dysfunctional physiological processes and, consequently, the onset of associated diseases.

### Phase separation regulates chromosome structure and cell division

Recent investigations have shed light on the pivotal role of LLPS in governing chromosome assembly and compartmentalization. Notably, Gibson et al. conducted studies demonstrating that recombined chromosomes in vitro exhibit characteristic LLPS behavior when immersed in a saline environment, giving rise to droplets dependent on histone tails. Intriguingly, the phase separation of chromosomes intensifies with an increasing number of nucleosomes. Within cellular contexts, histone H1 emerges as a significant factor in promoting the phase separation of chromosomes, thereby elevating the concentration of nucleosomes within the droplets. Conversely, the acetylation of histones contributes to the dissolution of these droplets [28]. Furthermore, it has been discovered that N-terminally phosphorylated heterochromatin protein 1α (HP1α) has the capacity to bind with histone H3 lysine 9 methyl (H3K9me3), forming droplet-like condensates. This interaction plays a crucial role in regulating nucleosome assembly and constriction of heterochromatin, consequently influencing gene silencing mediated by heterochromatin [29]. The HP1 protein Swi6 in fission yeast exhibits the ability to condense with nucleosomes, altering the conformation of histone octamers within nucleosomes and facilitating heterochromatin compression [30]. Notably, aberrant phase separation has been implicated in promoting cancer by modulating chromosome assembly. In instances of human leukemia, recurrent chromosomal translocations give rise to an aberrant chimeric cancer variant featuring NUP98 and HOXA9 proteins. This fusion, NUP98-HOXA9, instigates the formation of chromatin loops independent of CTCF, subsequently enhancing the expression of proto-oncogenes and culminating in the onset of leukemia [31].

Recent discoveries have unveiled the presence of phase-separated condensates within various structures crucial to cell division, including centrosomes, centromeres, and mitotic spindles. Phase separation emerges as a fundamental mechanism governing cell division by orchestrating the assembly of molecules involved in this intricate process [4]. For instance, end-binding protein 1 (EB1), driven by multivalent interactions among its distinct regions, interfaces with microtubules to initiate LLPS and form molecular condensates. EB1's involvement in regulating the dynamics of microtubule spindles significantly influences chromosome movement during mitosis. Extensive research has demonstrated that the LLPS of EB1 serves as the foundation for precise chromosome segregation during mitosis. Consequently, deficiencies in LLPS, as observed in EB1 mutants, give rise to defects in chromosome separation and errors in spindle localization, ultimately contributing to conditions such as cancer [32, 67]. By delving into the LLPS phenomena induced by specific proteins within MLO, we can unravel the intricate molecular mechanisms underpinning the formation of diverse intracellular compartments during mitosis, gaining insights into their associations with various diseases [68].

### Phase separation involves signaling pathway transduction and gene expression

Cell signaling pathways intricately rely on the formation of functional molecular condensates through phase separation, ultimately facilitating the progression of signaling cascades. A prime example of this phenomenon can be observed in the cGAS-STING signaling pathway, comprising key components such as cyclic GMP-AMP synthase (cGAS), stimulator of interferon genes (STING), and downstream signal adapters. Upon binding to cytoplasmic double-stranded DNA (dsDNA), cGAS triggers a cascade of events involving conformational changes and DNA-linked LLPS [33]. Crucially, the distinctive structure of the cGAS-DNA condensate significantly enhances the central role of LLPS in cGAS activation, resulting in a substantial augmentation of the efficiency of cGAS enzymatic reactions [69]. Recently, Li et al. demonstrated that Tumor necrosis factor receptor (TNFR)-associated factor 6 (TRAF6) undergoes phase separation in response to TLR4 signaling, and Suppressor of Fused (Sufu) functions through interfering phase-droplet formation of TRAF6, thereby restraining its signaling activity [34].

Phase separation plays a pivotal role in gene expression, with transcription-related molecules forming condensates to finely tune the activity of target genes. A striking example of this phenomenon is observed in peroxisome proliferator-activated receptor gamma (PPARγ), a member of the nuclear hormone receptor (NR) superfamily of proteins, which can assemble condensates for gene expression regulation [70]. When PPARγ is activated by ligand binding, it binds to retinoic acid X receptor (RXR)α to form a heterodimer. The complex orchestrates its binding to target genes through the mediation of a DNA binding domain characterized by the zinc finger motif. This interaction is directed towards the peroxisome proliferation response element (PPRE) located within the promoter region, initiating PPARγ-specific transcriptional activation. A noteworthy aspect of this process is that PPARγ forms nuclear condensates specifically at the PPRE site. This spatial compartmentalization effectively separates it from RXRα, which forms a separate heterodimer, thereby facilitating PPARγ's role in governing gene expression [35]. This regulatory mechanism extends its influence on various physiological processes such as glucose and lipid metabolism, the inflammatory immune response, and more [71]. As a pivotal mechanism, phase separation plays a crucial role in regulating the expression of PPARγ target genes. However, it does not operate in isolation; instead, it synergizes with other traditional mechanisms of PPARγ action, such as the recruitment of co-activators and co-repressors [72]. Consequently, it becomes imperative to enhance our comprehension of the interplay between phase separation and the expression of target genes within the PPARγ pathway.

### Phase separation participates in autophagy process

Phase separation plays a pivotal role in regulating autophagy, influencing various physiological activities in organisms. This phenomenon involves the condensation of ATG proteins, leading to the assembly of pre-autophagosomal structures (PAS), autophagosome formation, and the subsequent initiation of autophagic degradation. The Atg1 complex, responsible for initiating autophagy, induces phase separation through Atg13 and Atg17's multivalent interactions, facilitating PAS liquid condensate assembly [36]. Furthermore, TORC1 kinase activity regulates PAS assembly via Atg13 phosphorylation. In nutrient-rich conditions, hyperphosphorylated Atg13 inhibits Atg1 complex formation, thus preventing PAS assembly. Conversely, nutrient deprivation or rapamycin treatment dephosphorylates Atg13, promoting PAS assembly [7]. Under certain stress or pathological conditions, LLPS can undergo an abnormal phase transition, shifting from a liquid to a gel state. This transition can disrupt autophagic degradation, resulting in abnormal protein condensate accumulation, which is associated with conditions like cancer, neurological diseases, and cardiovascular diseases [73]. For instance, during heat stress, PGL-1 and PGL-3 proteins in nematode embryonic somatic cells evade autophagic degradation, leading to abnormal accumulation of protein condensates known as PGL particles. These PGL particles recruit mRNA and RNA-binding proteins, initiating LLPS to form liquid biomolecular condensates. mRNA promotes PGL protein phase separation, inhibiting abnormal phase transitions by scaffold protein EPG-2, ultimately hindering PGL particle autophagic degradation [37].

Additionally, phase separation has significant roles in regulating cytoskeletal assembly and synapse formation. Research has revealed that the IDR of microfilament-binding protein abLIM1 can induce LLPS to form condensates, whereas its LIM domain inhibits phase separation. Consequently, abLIM1 mutants with defective LIM domains can enhance microfilament nucleation and bundle microfilaments through LLPS [38]. In the nervous system, synaptic Arc proteins respond to neuronal stimulation by influencing the formation of postsynaptic density (PSD) condensates and aggregation of AMPA receptors (AMPAR). High Arc concentrations in synapses inhibit PSD-95 and the auxiliary subunit TARPs of AMPAR from undergoing phase separation. Dispersing TARPs from PSD condensates leads to AMPAR downregulation via endocytosis, resulting in weakened synapses [39].

In summary, phase separation condensates play diverse physiological roles in the human body, serving as essential components for maintaining normal cell function and various biological processes. The polymerization and depolymerization of these condensates are intricately regulated, ensuring precise timing and spatial coordination of their functions. Consequently, delving into the formation and regulation of phase separation condensates contributes to an improved understanding of intricate life activities and facilitates exploration into the intricate relationship between biomolecular condensates and various diseases.

### Driving forces of phase separation

Phase separation of biomacromolecules is primarily governed by polyvalent interactions. Within cells, this force can be orchestrated by proteins containing multiple modular domains with similar functions, IDRs, RNA, and more [74].

Proteins featuring multiple modular domains with similar functions exert control over phase separation through specific interactions between these modules. For instance, the SRC homology 3 (SH3) and proline-rich motif (PRM) represent a common interacting pair, and research has identified LLPS involving tandem repeats of SH3 and PRM [40]. Notably, efficient LLPS necessitates a multivalent interaction force between highly valent polymer sequences, such as ((SH3) n) and ((PRM) n). However, Hong et al. demonstrated the induction of protein condensates with minimal scaffold modules using a divalent metal ion-single-component scaffold (6 His). In this context, a straightforward fusion of 6His-labeled SH3 and PRM proteins can undergo LLPS and form protein condensates [75].

In addition to conventional structural domains, certain proteins encompass regions lacking a fixed structure and stable tertiary conformation, referred to as IDRs or low complexity regions (LCRs) [76]. IDRs are frequent sites for phase separation, characterized by low sequence complexity and comprising specific amino acid types, including hydrophilic (serine, arginine, glutamic acid, and lysine), aromatic (tyrosine, phenylalanine, and tryptophan), and charged amino acids. The presence of IDRs facilitates the formation of weak multivalent interactions such as π-π interactions, cation-π interactions, cation–anion interactions, dipole–dipole interactions, electrostatic interactions, and hydrophobic interactions between protein molecules [77], rendering proteins more susceptible to phase separation. For example, DDX4's IDR is enriched in phenylalanine-glycine repeats, which drive phase separation [41].

Phase separation occurs not only among proteins but also between proteins and nucleic acids [78]. Wang et al. discovered that certain proteins not only possess IDRs but also include RNA-binding domains (RBDs). RBDs consist of one or more folded RNA recognition modules and include IDRs. These disordered regions within RBDs frequently exhibit a high abundance of glycine and positively charged residues, such as arginine. It has been established that the phase separation of FUS family proteins, which feature both IDRs and RBDs, primarily hinges on multivalent interactions between tyrosine residues in the protein-like region and arginine residues in the RNA-binding region [42]. Shen et al. observed that NLRP6 (Nucleotide-Binding Oligomerization Domain-Like Receptor Pyrin Domain-Containing Protein 6) can directly interact with double-stranded RNA (dsRNA) to create a dynamic liquid condensed phase. Activated NLRP6 can induce inflammasome activation, pyroptosis, and interferon production, thereby influencing intestinal homeostasis, and microbial colonization, and playing a pivotal role in various infectious diseases and inflammation [44].

Interestingly, interactions solely among RNAs can also drive phase separation, leading to the formation of RNA condensates, such as SGs and Processing bodies (P bodies) in mammalian cells [27]. RNA-RNA interactions exert a robust multivalent force, and nearly any pair of RNAs can interact rapidly to form polymers. This multivalent interaction force becomes more pronounced with increasing RNA concentration, thereby facilitating the creation of "droplets." Philip et al. revealed that RNA-RNA polymers can be composed of as few as 4 base pairs of RNA fragments, encompassing ring-ring RNA base pairing and RNA sequences with CNG repeats. RNA aggregation is more likely to occur among multiple chains [79].

Furthermore, phase separation is influenced by concentration and macromolecular crowding. When the concentration of specific proteins or RNA molecules surpasses their solubility threshold, they can undergo LLPS, transitioning from the solution into droplets. With increasing concentration, the significance of multivalent weak interactions between proteins and RNA is magnified, facilitating the formation of these "droplets" [80]. Within cells, a diverse array of macromolecules populates a highly crowded environment. This molecular crowding fosters interaction between proteins and RNA within the droplets, consequently promoting phase separation and impacting droplet properties such as viscosity and fusion reactions [81]. Simultaneously, phase separation is subject to regulation by various physiological and pathological factors, including temperature, pH, ATP/energy levels, and ionic strength. Manipulating these influential parameters can control the formation and dissolution of biomolecular condensates arising from phase separation. This underscores phase separation as a dynamic and reversible process capable of achieving the aggregation and depolymerization of biomolecules through alterations in environmental conditions. Notably, many proteins exhibit heightened sensitivity to pH changes, with even minor pH fluctuations triggering phase separation [82]. Environmental conditions can thus modulate the propensity and stability of biological macromolecule droplets, while changes in cell physiology or stress responses can either initiate or disrupt the phase separation process [83].

To encapsulate, phase separation emerges as a sophisticated biophysical phenomenon influenced by factors such as multivalent interactions among biological macromolecules, concentration, macromolecular crowding, temperature, and pH. These elements synergistically induce the aggregation of biological macromolecules within cells, contributing to a spectrum of biological processes. Notably, these condensates are subject to simultaneous regulation by a diverse array of effects.

### Regulation of phase separation

The dynamic and reversible aggregation and disaggregation of liquid molecular condensates formed through LLPS are intricately regulated by a multitude of mechanisms. These include post-translational modifications (PTMs), interactions with membrane surfaces, involvement of molecular chaperones, the activity of RNA helicases, and the modulation of RNA itself, among others. By modulating the driving forces behind phase separation, the generation of biomolecular condensates can be either facilitated or impeded, thereby enabling precise control over phase separation. Disruption in the regulation of phase separation can result in the formation of aberrant condensates, consequently triggering the onset of related diseases.

### PTMs and phase separation

PTMs play a pivotal role in the regulation of protein phase separation by inducing conformational alterations through processes such as phosphorylation, ubiquitination, glycosylation, methylation, and acetylation. These modifications can modulate intermolecular interactions, thereby promoting or inhibiting the occurrence of phase separation [84].

Among PTMs, phosphorylation emerges as the most prevalent type affecting protein LLPS. This effect is characterized by its rapid and reversible nature. Phosphorylation can either facilitate or impede biomolecular condensation in diverse cellular contexts. For instance, Wegmann et al. identified over 80 potential phosphorylation sites within the amino acid sequence of the pathological condensate Tau protein associated with AD, with phosphorylation being its predominant PTM. LLPS of Tau is primarily driven by electrostatic and hydrophobic interactions. Phosphorylation introduces negative charges to the proteins, altering the charge distribution and electrostatic interactions along the polypeptide backbone. This, in turn, promotes the phase separation and aggregation of Tau [24]. Conversely, Lara et al. discovered that excessive phosphorylation of the C-terminal serine residue of TDP-43, a major protein condensate detected in amyotrophic lateral sclerosis (ALS) and frontotemporal dementia (FTD), inhibits its own phase separation and condensation. The researchers hypothesize that hyperphosphorylation of TDP-43 may serve as a protective cellular mechanism countering the abnormal phase transition of TDP-43. This is achieved by reducing multivalent interaction forces at the C-terminus through negatively charged and highly hydrated phosphate groups. Consequently, TDP-43 becomes more dynamic and mobile. These findings highlight that abnormal PTMs observed in pathological condensates may not universally act as drivers of protein condensation but can also fulfill a protective, anti-aggregation role [45].

Ubiquitination, a process catalyzed by a signal cascade involving ubiquitin-activating enzymes (E1), ubiquitin-conjugating enzymes (E2), and ubiquitin-ligase enzymes (E3), results in the covalent attachment of ubiquitin to target proteins [85]. Research indicates that ubiquitination plays a crucial regulatory role in the phase separation of chromatin and various nuclear structures [86]. Some ubiquitin-related molecules have been identified as participants in the regulation of biomolecular condensate formation or membraneless organelles created through specific biomolecular LLPS, including SGs, nuclear speckles, and autophagosomes [87]. Ubiquitination can exert both positive and negative regulatory effects on protein phase separation. For instance, Sun et al. demonstrated that the addition of K63 polyubiquitin chains to the scaffold protein P62 induces the phase separation of P62, leading to the formation of biomolecular condensates of P62 bodies. This polyubiquitin-dependent phase separation of P62 occurs through the PB1 domain, which interacts with ubiquitin via the UBA domain. Furthermore, the study revealed that phosphorylation of S403 enhances the formation of P62 bodies [46]. Dao et al. demonstrated that the ubiquitin adapter protein UBQLN, involved in the protein quality control(PQC) mechanism, can interact with ubiquitin molecules on ubiquitinated substrate proteins through its UBA domain. Specifically, ubiquitination can inhibit LLPS within SGs under stress conditions by disrupting the weak multivalent interaction between UBQLN2 and its substrate. Simultaneously, UBQLN2 transports ubiquitinated substrate proteins within SGs to the proteasome for degradation through its UBL domain [47].

Glycosylation stands as another PTM with a crucial role in the regulation of phase separation [88]. Research has illuminated the capacity of O-acetyl-glucosamine glycosylation (O-GlcNAcylation) modification to govern the aggregation of certain proteins linked to neurodegenerative disorders, such as α-synuclein (α-syn) and Tau protein. O-GlcNAcylation occurs on serine and threonine side chains, facilitated by O-GlcNAc transferase (OGT), and reversed by O-GlcNAcase enzyme (OGA). Importantly, phosphorylation can co-occur with O-GlcNAcylation, with O-GlcNAcylation affecting phosphorylation. This interplay can potentially reduce the concentration of α-syn and Tau protein condensates, thereby offering insights into the progression of diseases like Parkinson's and Alzheimer's [48]. Furthermore, O-GlcNAcylation has extensive interactions with other PTMs, including acetylation, methylation, and ubiquitination [89].

Other PTMs can also modulate protein phase separation by perturbing cation-π interactions. Examples include arginine methylation (inhibition), arginine citrullination (inhibition), and lysine acetylation (inhibition/promotion) [84]. It is noteworthy that the phase separation process of a given protein can involve the combined effects of multiple PTMs. Thus, investigating the mechanisms by which PTMs interact can enhance our understanding of how PTMs regulate protein phase separation and their roles in the development of various diseases.

### Membrane surface interaction and phase separation

The phase separation of biological macromolecules typically occurs in three-dimensional solutions via multivalent interactions, and emerging research suggests that the cell membrane surface plays a crucial role as a regulatory platform for orchestrating the controlled assembly of biomolecular condensates. The membrane surface confines protein diffusion to a two-dimensional plane, fostering the aggregation of biological molecules by creating micron-sized protein clusters on the membrane. This phenomenon effectively lowers the molecular threshold concentration required for phase separation, facilitating protein aggregation on the membrane surface and culminating in two-dimensional phase separation [90]. Consequently, once signaling proteins are activated in various signaling pathways, they amplify signal transmission through phase separation, culminating in the assembly of protein clusters on the membrane [91]. For instance, the Nephin-NCK-N-WASP system, which governs actin polymerization in renal podocytes, promotes actin assembly on the membrane surface [49]. The SH2 domain within NCK interacts with the phosphotyrosine residues in Nephin, while its SH3 domain engages with the proline-rich domain of the actin regulatory protein N-WASP. This interaction results in the formation of Nephin-NCK-N-WASP protein clusters on the membrane. In the presence of actin and the N-WASP target Arp2/3 complex, this protein cluster induces actin polymerization, thus regulating the actin signaling pathway [92]. Additionally, during T cell receptor (TCR) activation, the T cell activation linker protein (LAT) plays a pivotal role as a transmembrane protein connector. When TCR activation occurs, LAT undergoes phosphorylation, leading to the formation of micron-sized clusters on the membrane alongside proteins Grb2 and Sos1. Grb2 and Sos1 are essential for this protein cluster's formation. Grb2's SH2 domain binds to phosphotyrosine in LAT, while its SH3 domain interacts with the proline-rich domain in Sos1. Upon binding to the membrane, Sos1 undergoes a conformational change, promoting RAS activation and the conduction of downstream signals [50]. LAT clusters mediate crucial cellular processes, including calcium mobilization, mitogen-activated protein kinase (MAPK) activation, and the facilitation of biochemical reactions within downstream signaling pathways. This particularly includes the promotion of NCK-N-WASP-Arp2/3-mediated actin polymerization [93].

Significantly, the control of condensates extends beyond the surface of the plasma membrane to include the inner membrane. Notably, the ER membrane holds a crucial role in this regulatory landscape [94]. Recent discoveries highlight that AGO proteins, in association with miRNAs, undergo lipid-mediated phase separation on the ER membrane, coalescing into RNP particles that control protein production. This condensation is orchestrated by electrostatic interactions between the conserved lipid-binding motif in AGOs and the lipid PI (4,5) P_2_. This interaction recruits the E3 ubiquitin ligase Ltn1 to catalyze the ubiquitination of nascent polypeptides, collaborating with the VCP-Ufd1-Npl4 complex to facilitate proteasomal degradation [51]. Moreover, the ER membrane plays a significant role in governing the assembly of autophagy initiation sites during autophagy. Stimulation of autophagy triggers Ca^2+^ oscillations on the outer surface of the ER membrane, promoting the formation of LLPS and droplet-like condensates by the autophagy-initiating FIP200 complex on the ER membrane. These condensates interact with endoplasmic reticulum membrane proteins VAPA/B and ATL2/3, localizing them on the ER and serving as autophagy initiation sites [52]. Additionally, other organelle membranes, such as endosomal membranes [95] and lysosome membranes [96] may also play a significant role in regulating the formation of biomolecular condensates.

### Molecular chaperones and phase separation

Molecular chaperones represent a class of proteins dedicated to facilitating intracellular molecular assembly and protein folding, integral to the maintenance functions of diverse proteomes, including prominent members such as Hsp60, Hsp70, and Hsp90 [97]. Numerous studies have demonstrated that molecular chaperones play a pivotal role in modulating the phase separation of biological macromolecules. These chaperones can either facilitate or impede phase separation, ensuring the regulated localization and temporal coordination of these macromolecules within cells. For instance, transporter protein1 (TNPO1) has been identified as a molecular chaperone for FUS protein, acting at both the distal axonal compartment and nuclear pores of neurons to modulate FUS phase separation. In cases of abnormal FUS accumulation, TNPO1 functions to inhibit the formation of FUS granules [43]. Chaperones Hsc70 and Hsp90 collaboratively act as molecular chaperones on the amino terminus and Tyr39 residue of α-syn to prevent its aggregation. Inhibitors of Hsc70 and Hsp90 disrupt this protective effect, leading to α-syn reaggregation. Experimental evidence demonstrates that the interaction between Hsc70 and Hsp90 regulates α-syn accumulation, a key factor in Parkinson's disease development. This finding opens new avenues for potential treatments targeting α-synucleinopathies [98]. These studies collectively illustrate the critical role of molecular chaperones in preserving protein function and mitigating neurodegenerative diseases arising from aberrant neuronal protein deposition. Molecular chaperones accomplish this by preventing the aggregation of misfolded proteins, regulating abnormal phase separation, and facilitating the dissolution of deleterious aggregates. These insights highlight the potential of molecular chaperones as intervention targets for degenerative proteinopathies [99].

### RNA and phase separation

RNA helicases, as energy-consuming proteins, actively participate in the regulation of biomolecular condensate formation by reorganizing RNA structures. Studies have highlighted the importance of the ATP-dependent DEAD-box protein family of RNA helicases in various RNA metabolic processes, playing a significant role in genome stability and the pathogenesis and treatment of tumor [100, 101]. DEAD-box RNA (DDX) helicases localize to SGs and function as pivotal regulators of SGs and other ribonucleoprotein condensates [102]. For instance, recent research has unveiled that both DDX helicases DDX3X and DDX3Y, encoded by sex chromosomes, can promote the LLPS of FUS protein. Interestingly, DDX3Y exhibits greater efficacy in inhibiting mRNA translation and promoting FUS protein condensation compared to DDX3X. This discovery offers fresh insights into investigating gender-related disparities in RNA metabolism and their implications for human diseases [103].

Additionally, RNA molecules themselves can act as regulators, participating in the control of the phase separation process. Research suggests that the phosphate backbone of RNA inherently imparts a high negative charge density, thereby influencing the state of condensates formed through the phase separation of biological macromolecules during the transcription process, primarily through electrostatic interactions. At low RNA concentrations, RNA appears to promote interactions between opposite charges, fostering condensate formation. Conversely, at high RNA concentrations, RNA appears to discourage interactions between like charges, leading to condensate dissolution [104, 105]. Furthermore, RNA's composition, length, structure, and modifications can impact the composition, size, shape, viscosity, fluidity, surface tension, and other characteristics of biomolecular condensates [106].

**Fig. 1:**
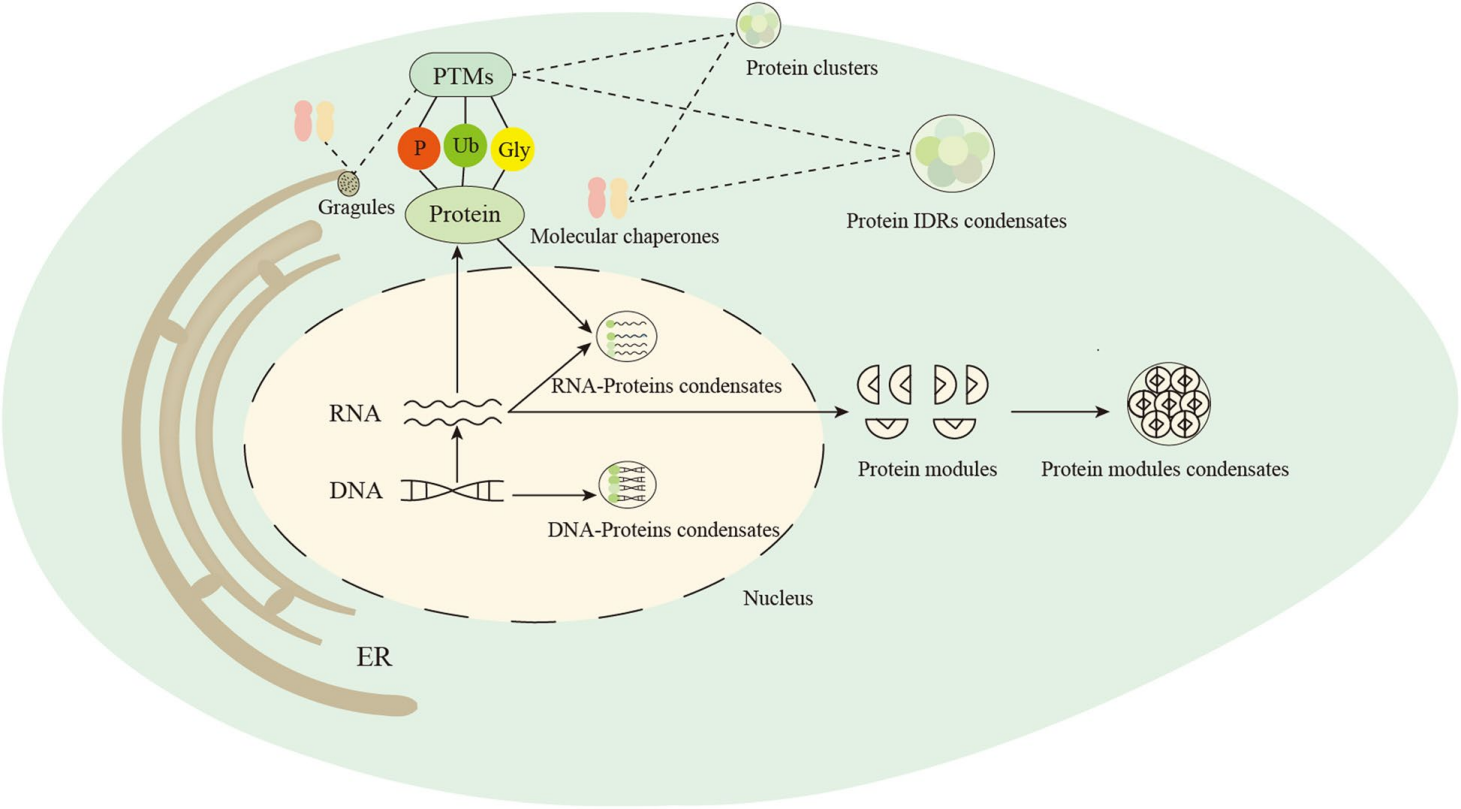
Formation and regulatory mechanisms of biomolecular condensates. RNA, transcribed from DNA, transits the nuclear pores to enter the cytoplasm, where it undergoes translation into proteins possessing either IDRs or multiple modular domains with analogous functions within the nucleus. Proteins with IDRs can form condensates with target proteins in the cytoplasm or, upon entering the nucleus through nuclear pores, participate in the formation of condensates with specific DNA and RNA. Conversely, proteins with specific modules assemble condensates in the cytoplasm through interaction forces between these modules. The endoplasmic reticulum and the cell membrane contribute to the creation of surface protein condensates, such as protein granules and clusters, through interaction forces on the membrane surface. The intricacies of aggregation and disaggregation of protein condensates, protein granules, and protein clusters with IDRs in the cytoplasm are finely regulated by PTMs and molecular chaperones

To summarize, the regulation of phase separation encompasses various intricate mechanisms (Fig. 1). These regulatory processes collaborate to guarantee that phase-separated biomolecular condensates effectively execute normal biological functions within cells and appropriately respond to external stimuli and environmental changes. Among these mechanisms, PTMs play a critical role in phase separation regulation, with several other mechanisms interacting with PTMs. For instance, PTMs such as phosphorylation can govern the activation of Nephin and LAT proteins, thereby regulating their phase separation on membrane surfaces. PTMs can also collaborate with molecular chaperones and RNA helicases to modulate phase separation. Dysregulated control of phase separation can result in the abnormal aggregation of functional molecules, disrupting their normal physiological functions and fostering tumor development. In the case of LLPS, it undergoes a liquid-to-solid phase transition (LSPT), causing the irreversible transformation of liquid condensates into pathogenic amyloid aggregations. This transformation adversely impacts the function of nerve cells, triggering a range of neurodegenerative diseases [107]. Hence, by studying the intricate coordination of these mechanisms in regulating phase separation and preserving the homeostasis of biological macromolecules, we can better harness the potential of biomolecular phase separation for regulating abnormal condensates associated with diseases, thus playing a vital role in disease treatment.

### Phase separation and tumor

**Fig. 2:**
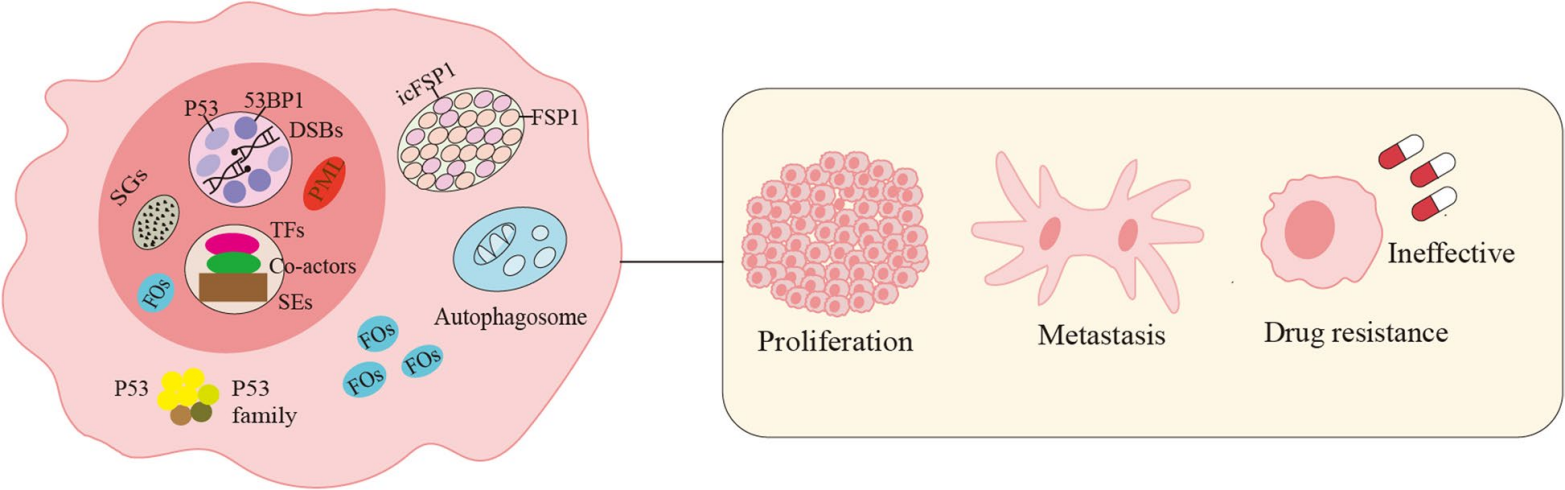
Biomolecular condensates in tumor. Within the cellular milieu of tumors, an array of biomolecular condensates manifests. In the nucleus, these encompass SGs, FOs, PML bodies, protein condensates associated with DSBs formed by P53 and 53BP1, along with TFs, co-activators, and SEs-related transcriptional condensates. In the cytoplasm, condensates are constituted by P53 and the P53 family proteins, FOs, autophagosomes, and Iron death-related condensates formed by icFSP1 and FSP1. The presence and dynamics of these condensates significantly influence tumor proliferation, metastasis, and the development of treatment resistance

Phase separation serves a multitude of vital physiological functions, encompassing the regulation of chromosome structure, cell division, signal transduction, gene expression, and DNA repair, and its significance extends to the preservation of cellular homeostasis. Abnormal phase separation can trigger the pathological aggregation of functional molecules, subsequently leading to associated diseases (Fig. 2). In recent years, an increasing body of evidence has highlighted the significant involvement of aberrant LLPS in tumor cells. This phenomenon plays a crucial role in the initiation, progression, and resistance to treatment of tumors by influencing DNA repair, transcriptional regulation, the expression of proto-oncogenes and tumor suppressor genes, as well as the formation of PML bodies and SGs. At the same time, targeting biomolecular condensates driven by abnormal phase separation in tumor represents a promising avenue for investigating tumor pathogenesis and identifying potential therapeutic targets.

### Phase separation participates in the DNA repair of tumor cells

The influence of phase separation extends to DNA repair processes, a critical component of the DNA damage response (DDR), which encompasses complex cellular reactions triggered by DNA damage. Dysregulation of DDR can lead to genome instability, contributing to tumorigenesis. Among the primary pathways for repairing DNA double-strand breaks (DSBs) are non-homologous end joining (NHEJ) and homologous recombination (HR) [108]. Emerging evidence suggests that LLPS plays a role in regulating DNA damage repair, and its close association with the occurrence and treatment of tumor is becoming increasingly apparent.

In the context of the DDR, studies suggest that numerous DDR proteins aggregate within the nucleus. This aggregation is facilitated by multivalent cooperative interactions in areas of protein and/or nucleic acid binding, along with IDRs. The resulting structure is reversible and forms a biomolecular condensate, which undergoes disassembly upon the completion of the repair process [109]. The activation of DDR at DNA break sites is initiated by the ATM kinase, which phosphorylates the histone variant H2A.X, generating γH2AX. This process induces the LLPS of DDR proteins along with damage-induced long non-coding RNAs (dilncRNAs). Among these, 53BP1 (p53-binding protein) is a principal component of functional condensates within DDR. Positioned at the double-strand break (DSB) site, 53BP1 exhibits high liquid mobility and plays a pivotal regulatory role in two DNA repair pathways. Specifically, it impedes DSB end resection in the G1 phase, promotes the DSB repair pathway mediated by NHEJ, facilitates the reconnection of DSB ends, and counters the initiation of HR [53, 110]. Subsequent studies have revealed the enrichment of DNA damage-induced 53BP1 droplets with the tumor suppressor protein P53. Disrupting the conditions conducive to 53BP1 phase separation inhibits the expression of P53 target genes and the 53BP1-dependent induction of P53. This suggests a potential novel strategy for targeted cancer therapy by interfering with this mechanism [111]. Much like the condensate formed by 53BP1, SLX4 also creates nuclear condensates in collaboration with the structure-specific endonuclease XPF. This interaction serves to recruit DDR factors, thereby enhancing DNA repair through HR. Additionally, the SLX4-XPF condensate exhibits interactions with SUMO-SIM, promoting its assembly on chromatin. This, in turn, facilitates the SUMOylation or SUMO-dependent ubiquitination of substrate proteins, expediting protein turnover at DNA damage sites and thereby contributing to the efficiency of DNA repair processes [54, 112]. In addition, during the DDR process induced by tumor radiotherapy, it was found that the overexpression of the NONO protein can lead to the formation of heterodimers with SFPQ that undergo LLPS. This process enhances the interaction between the nuclear translocation of EGFR and DNA-PK and the phosphorylation of DNA-PK, ultimately facilitating DNA repair mediated by the NHEJ pathway and tumor radioresistance. Experiments have underscored that the NONO protein is integral to DNA repair and radioresistance in tumor cells. Its overexpression fosters the formation of condensates driven by LLPS, bolstering DNA repair capabilities and radioresistance of tumor cells. Conversely, knockout of the NONO gene or use of the LLPS inhibitors targeting NONO can selectively target DNA repair and restore the sensitivity of tumor cells to radiotherapy, representing a pivotal strategy for enhancing the effectiveness of tumor radiotherapy [55, 113].

Chromosomal rearrangements arising from DNA breaks and misjoining at incorrect locations can result in the expression of fusion oncoproteins (FOs), recognized as oncogenic contributors in various cancers [114]. Currently, numerous FOs are identified as droplet-shaped biomolecular condensates formed through LLPS, comprising two primary types of phase-separated FOs. One type involves the generation of nuclear condensates, exerting carcinogenic effects by influencing the expression of chromatin-related genes. Notably, examples like NUP98-HOXA9 in AML and EWS-FLI1 FOs in Ewing sarcoma induce abnormal gene expression by forming chromatin-associated nuclear condensates, thereby promoting cancer development. The other type involves the formation of cytoplasmic condensates that facilitate the propagation of abnormal signals, such as RAS/MAPK signals, thus contributing to cancer onset. For instance, the driver EML4-ALK in lung cancer and the driver CCDC6-RET FOs in thyroid cancer can generate cytoplasmic condensates, promoting aberrant Ras signaling [56, 57]. These droplets steer cells toward a cancerous state by sequestering crucial protein and RNA molecules. In the future, therapeutic drugs targeting oncogenic fusion oncoproteins can be designed to specifically counteract the tumor pathology instigated by these ectopic condensates [115]. It's noteworthy that certain studies have introduced a high-throughput screening program named DropScan, enabling the analysis and screening of FOs formed through various abnormal condensation events. This facilitates the discovery of small molecule modulators, holding significant implications for the investigation of FOs-related cancer treatment [116].

### Phase separation is involved in transcriptional regulation and oncogene expression

The development and progression of tumor are accompanied by transcriptional dysregulation. During transcription, transcription factors (TFs) regulate gene expression by recognizing and binding to specific genomic sequences [117]. Enhancers, particularly super-enhancers (SEs), play a pivotal role in gene transcription regulation. SEs denote transcriptional regulatory complexes resulting from the cooperative assembly of transcription factors, transcription co-factors, chromatin regulators, non-coding RNAs, and RNA polymerase II (RNAPII). In contrast to individual enhancers, SEs exhibit heightened efficacy in governing gene transcription. The current understanding affirms that both enhancers and SEs are membraneless organelles formed through phase separation [58]. In the context of cancer, SEs can drive elevated intracellular expression of oncogenes and assume a crucial role in boosting the transcription of proto-oncogenes upon which cancer cells heavily rely [118]. The mechanism is that IDRs-containing TFs, transcription coactivators, and RNAPII can undergo phase separation on the target genes of super enhancers, consequently enhancing the expression of related transcriptional regulatory elements [119].

Research has demonstrated that, under conditions of hyperosmotic stress, the transcriptional coactivator YAP undergoes LLPS in both the nucleus and cytoplasm, resulting in the formation of liquid condensates. These condensates facilitate the redistribution of YAP into the nucleus. Within the nucleus, YAP condensates are enriched with YAP-specific transcription factors and co-activators, activating the transcription of target genes and thereby fostering cell proliferation and differentiation [120]. The Hippo signaling pathway, functioning as a tumor suppressor pathway, regulates the cellular localization of YAP/TAZ. Upon deactivation, it allows YAP and TAZ to accumulate in the nucleus, leading to an elevation in gene expression levels. Furthermore, this process promotes the binding of YAP to DNA-binding cofactor TEAD, transcription coactivators BRD4 and MED1, and transcription elongation factor CDK9 on SEs, resulting in the formation of a phase-separated biomolecular condensate. This condensate activates the transcription of proto-oncogenes, thereby promoting tumorigenesis [59]. For instance, in lung cancer, YAP forms condensates through interaction with TEAD and SRC-1, widely enhancing the transcription of YAP in cancer cells and consequently promoting the growth of YAP-dependent cancer cells [121]. In a mouse lung adenocarcinoma model, interferon-γ (IFN-γ) triggers the phase separation of YAP, conferring resistance to anti-PD-1 immunotherapy in tumor cells. Disrupting YAP phase separation inhibits tumor growth, enhances immune responses, and restores tumor cell sensitivity to anti-PD-1 treatment [122].

Moreover, proteins such as FUS, EWS, and TAF15, which incorporate low complexity domains (LCDs), have the capacity to form condensates through phase separation with the C-terminal domain (CTD) of the RPB1 subunit of RNA polymerase II (RNAPII) [123]. This interaction results in the activation of the expression of pertinent proto-oncogenes, a phenomenon evidenced in myxoid liposarcoma and Ewing's sarcoma [124]. Interestingly, Liu et al. found in a recent study that glycogen accumulation is commonly present in the early stage of human and mouse liver tumors and essential for tumor initiation. The accumulated glycogen in tumor cells undergoes LLPS, which leads to the assembly of the Laforin-Mst1/2 complex in glycogen liquid droplets and consequently activates Yap for cell survival and transformation [60].

In tumor, certain proteins or RNAs undergo phase separation and directly contribute to the abnormal activation or overexpression of proto-oncogenes, as well as the compromised activity of tumor suppressor genes, thus fostering tumor development. For instance, a long non-coding RNA (LncRNA) known as Kb-1980E6.3 exhibits abnormal upregulation in clinical breast cancer tissues induced by hypoxia. This LncRNA can associate with insulin-like growth factor 2 mRNA binding protein 1 (IGF2BP1) to form condensates, amplifying the stability of c-myc mRNA under hypoxic conditions. Consequently, this process promotes the self-renewal and tumor formation of breast cancer stem cells (BCSCs) [61]. Furthermore, research has demonstrated that a cluster of mutations in the tumor suppressor gene p53 is prevalent in most cancers. These mutations result in the loss of tumor suppressor p53 function, thereby promoting cancer development. Mutant p53 aggregates encompass both the abnormal self-aggregation of p53 and the co-aggregation of its mutants with p53 family proteins [62]. Intriguingly, p63 and p73 have been shown to co-aggregate with mutant p53, forming biomolecular condensates that acquire negative oncogenic activity. This suggests that impeding the formation of p53 condensates may reinstate its capability to curtail excessive cancer cell proliferation and foster cell death, offering intervention strategies for mitigating p53 mutation aggregation [125]. Understanding the impact of phase separation on the expression of proto-oncogenes and tumor suppressor genes could unveil novel therapeutic approaches for tumor treatment. Presently, various studies are investigating ways to regulate the expression of tumor-related genes by intervening in the phase separation process. This may involve the use of drugs to impede the formation of phase separation or disrupt the formed phase separation structure. Such interventions aim to inhibit the overexpression of proto-oncogenes or enhance the function of tumor suppressor genes.

### Phase separation affects the formation of PML bodies

PML bodies, also known as nuclear bodies (NBs), are membraneless organelles formed through phase separation. They are localized within specific areas of the nucleus, bind to specific functional proteins, and participate in the regulation of various cellular processes, including cell proliferation, apoptosis, DNA repair, transcriptional regulation, tumorigenesis, and more [126]. The protein Death domain-associated protein (DAXX) was identified within PML bodies (PML-NBs). DAXX undergoes phase separation and condensation into droplets facilitated by the polyvalent interaction of its SIM motif with SUMO. SPOP, a tumor suppressor, promotes the degradation of DAXX within these droplets. This action inhibits the overexpression of DAXX in tumor cells, thereby disrupting its capacity to promote tumor formation, proliferation, metastasis, and resistance to treatment when highly expressed [127, 128]. Additionally, PML-NBs play a role in depositing the histone variant H3.3 at specific chromatin sites and facilitating PTMs that interact with other biomolecules [129, 130]. DAXX protein needs the help of PML-NBs condensate in transcription regulation, apoptosis, viral infection, and tumorigenesis [131].

PML-NBs are involved in the regulation of the cell cycle. For instance, overexpression of PML in HeLa cells can induce G1 phase arrest and S phase delay, effectively inhibiting cell growth [63]. In acute promyelocytic leukemia (APL), chromosomal translocation leads to the formation of a heterodimer (PML-RARα) between PML and retinoic acid receptor α (RARα). This interaction disrupts the PML bodies structure and diminishes the transcriptional function of RARα, consequently impeding myeloid cell differentiation [64]. Additionally, PML-RARα acts as a transcriptional repressor, diminishing the transcriptional activity of RARα and thereby impeding myeloid differentiation [132]. Furthermore, the interference of PML-RARα with PML and RARα functions leads to the condensation of DSB repair proteins and delays the activation of ATM protein, resulting in DNA damage [133]. Some studies have also identified the mutant A216V of PML-NBs, which disrupts its LLPS. Consequently, this reduces PML's ability to recruit partner proteins like DAXX and UBC9 and affects post-translational modifications such as SUMOylation, phosphorylation, and acetylation of PML. Ultimately, eventually leading to impairment of the PML network function and resistance to arsenic-targeted therapy for leukemia [134].

### Phase separation regulates the formation of stress granules

When cells encounter stressors such as oxidative stress or chemotherapy drugs, phase separation occurs between mRNA and RNA-binding proteins through multivalent interactions, forming liquid biomolecular condensates known as SGs. Extended or aberrant SGs formation can contribute to alterations in the biological behavior of tumor cells [135]. SGs play a pivotal role in regulating the survival of tumor cells under stress conditions, influencing cell proliferation, invasion, metastasis, and chemoresistance, while also inhibiting apoptosis by modulating gene expression and signal transduction processes. Simultaneously, in the context of disruptions in Ras, mTOR, and histone deacetylase 6 (HDAC6), among others, SGs can promote tumor signaling by enhancing translation and the assembly of core components of protein–protein interactions within the SG structure. This promotes the formation of SGs within cancer cells in response to stress, augmenting the adaptability of cancer cells [136]. These findings suggest that SGs may function as a pivotal regulator and potential therapeutic target in cancer.

The G3BP1 protein acts as a core component in the assembly of SGs and can regulate LLPS through interactions involving its three distinct IDRs, thereby controlling SG assembly [137]. In another study, it was found that SGs protein YB-1 is an oncoprotein that can directly bind to the non-coding region at the 5' end of G3BP1. This interaction results in the increased expression of G3BP1 protein, facilitates SGs assembly, and plays a role in regulating cancer cell growth and proliferation, thereby expediting tumor development. The overexpression of YB-1 is linked to cancer treatment resistance and an unfavorable prognosis [138]. Therefore, studies by Somasekharan et al. demonstrated that knocking out the YB-1 or G3BP1 genes in myeloma cells can reduce SG assembly, thereby inhibiting tumor invasion and metastasis [139]. El-Naggar et al. additionally demonstrated that a class I HDAC inhibitor can impede the binding of YB-1 to G3BP1 by promoting YB-1 acetylation and inducing oxidative stress. This outcome leads to diminished G3BP1 levels and a decrease in SG assembly, ultimately accomplishing the inhibition of sarcoma metastasis [140].

Moreover, phase separation manifests on certain signaling proteins within numerous signaling pathways. Aberrant LLPS in this context can result in the dysregulation of signaling pathways, compromise the proper functioning of tumor suppressor proteins, or induce the constitutive activation of oncogenic pathways. Consequently, this process contributes to the progression of cancer. Such oncogenic signaling pathways include P53, Wnt/β-catenin, Hippo, TGF-β, AMPK, and mTOR signaling pathways [141]. In summary, in tumor, phase separation participates in multiple physiological processes such as DNA damage repair, transcriptional regulation, signal transduction, and the formation of PML bodies and SGs. Abnormal phase separation plays a significant role in driving tumor initiation, progression, and resistance to treatment by giving rise to biomolecular condensates.

### Therapeutic potential of targeting biomolecular condensates in tumor

In recent years, an increasing body of research indicates that targeted intervention against biomolecular condensates holds promise as a potential strategy for tumor treatment, particularly for condensates associated with characteristic features of tumor. The dynamics of LLPS can be modulated by disrupting the forces within the core components through mutagenesis, and sites of condensation, such as IDRs, can be targeted to disassemble LLPS. Alternatively, molecular genetic interventions or the use of corresponding antagonists in cells can be employed to regulate the abundance or localization of proteins or RNA, restoring the physiological function of biomolecules and eliminating tumor triggers. For condensates that inhibit tumors, specific agonists can be designed to enhance their phase separation, achieving targeted therapeutic effects. Simultaneously, this approach can heighten the sensitivity of tumor therapy by mitigating drug exclusion within droplet compartments formed by specific proteins.

### Cross-regulation of membraneless organelles using ligand-receptors

Numerous studies have demonstrated that the interaction between ligands and receptors provides an avenue for utilizing corresponding agonists or antagonists to selectively target phase separation condensates, thereby achieving tumor intervention and concurrently enhancing the sensitivity of tumor treatment. For instance, ligands acting as PPARγ agonists can inhibit tumor development by activating PPARγ and forming biomolecular condensates with co-activators. PPARγ activation occurs through natural or synthetic ligands like ω-3 polyunsaturated fatty acids (PUFA) and thiazolidinediones (TZD). Upon activation, PPARγ forms a heterodimer with RXR, translocates to the nucleus, and binds to the PPRE of PPARγ target genes, initiating gene transcription and participating in the regulation of multiple gene expressions [142]. Studies specifically in breast cancer have revealed the significant role of ligand-bound PPARγ in inhibiting the growth, metastasis, and invasion of breast cancer cells, impacting cell cycle regulation, and influencing both endogenous and exogenous apoptosis across various breast cancer subtypes, including ER + /PR + , ER-/PR-, HER + , and triple-negative breast cancer cells [143]. Additionally, follow-up investigations have linked PPARγ nuclear condensates to a better prognosis in breast cancer patients, whereas patients with cytoplasmic condensates exhibit a poorer prognosis [144, 145]. CB11, a novel PPARγ agonist, has emerged as a potential anti-cancer drug in targeting therapy for non-small cell lung cancer (NSCLC), demonstrating efficacy in overcoming radioresistance in NSCLC [146]. In contrast, in liver cancer, elevated levels of PPARγ nuclear condensates are associated with resistance to immune checkpoint blockade therapy (ICB). Consequently, the combined administration of PPARγ antagonists and ICB therapy proves effective in inhibiting liver cancer resistance to ICB, mediating the normalization of the tumor microenvironment (TME), and contributing to cancer treatment [147]. Furthermore, in prostate cancer (PCa), the androgen receptor (AR) and the auxiliary coactivator MED1 can form condensates at SEs sites in androgen-dependent PCa cells, facilitating oncogenic gene transcription. The use of AR antagonists inhibits phase separation and transcriptional activity by targeting their interacting IDRs, thereby impeding cancer cell formation [65]. Additionally, studies have identified a small molecule compound, ET516, as a potential AR phase separation inhibitor. ET516 binds directly to the N-terminal domain (NTD) of AR, inhibiting the formation of AR condensates and effectively suppressing the growth of castration-resistant PCa cells, ultimately overcoming proliferation and resistance to antiandrogen therapy [148].

### Regulation on phase separation through the PTMs mechanism

PTMs play a pivotal role in regulating the formation of biomolecular condensates associated with tumors. By specifically targeting the key components of their functions, PTMs can either promote or inhibit the formation of these condensates, offering novel strategies for targeting tumors and augmenting therapeutic efficacy. For example, E3 ubiquitin ligases, key enzymes in the ubiquitination process, have emerged as crucial regulators of tumor immune checkpoints and immune pathways, holding great potential in tumor immunotherapy [149]. Ubiquitin activation is an ATP-dependent process where it binds to cysteine residues on E1. Activated ubiquitin is then transferred to the cysteine active site of E2 and binds to it. Finally, E3 ubiquitin ligases facilitate the binding of ubiquitin to the lysine residues of the target protein, resulting in protein ubiquitination [150]. Among the E3 ubiquitin ligase families, the Cullin-Ring E3 ubiquitin ligase (CRL3) stands out as the largest in the body, forming a multi-protein complex. Specifically, Cullin 3 (CUL3) can associate with adapter proteins containing BTB domains to serve as scaffold proteins, forming E3 ubiquitin ligase complexes that recognize and mediate the ubiquitin-proteasomal degradation of substrate proteins [151]. Studies have revealed that SPOP, a BTB domain-containing protein, mediates the interaction between CUL3 and substrates. The LLPS of substrates and ubiquitin ligases forms the basis for concentrating substrates into "droplets," which is crucial for maintaining intracellular protein balance [152]. Dysfunctions in CUL3 have been linked to various malignant tumors, suggesting that E3 ubiquitin ligases can be explored as new targets for targeted cancer therapy [153]. Moreover, combining E3 ubiquitin ligase-targeted therapy with ICB has demonstrated potential in enhancing anti-tumor immune activity and clinical efficacy. This combined approach can modulate the activation or inhibition of cancer-related immune pathways through the phase separation mechanism, ultimately influencing tumor development positively or negatively [154]. Furthermore, targeted inhibition of cGAS methylation presents a potential therapeutic strategy for cancer. cGAS can undergo LLPS with dsDNA in the cytoplasm, generating the second messenger cGAMP. This cGAMP activates the STING-TBK1-IRF3-IFN pathway, playing a crucial role in innate immune responses and anti-tumor immunity [155]. While most endogenous cGAS is localized in the nucleus, its activity is restrained by chromatin tethering and cell cycle-dependent phosphorylation [156, 157]. Fang et al. discovered that methylation modification mediated by the methyltransferase SUV39H1 enhances the chromatin tethering of cGAS and suppresses its anti-tumor activity in a UHRF1-dependent manner. By restricting methionine uptake or inhibiting SUV39H1, the methylation modification of cGAS can be curtailed, promoting its release from chromatin binding. This enables its relocation to the cytoplasm, where it forms condensates with dsDNA, thereby inhibiting the development of colorectal cancer and augmenting the efficacy of radiotherapy and ICB [158].

### Regulation on phase separation associated with promoting cell death pathways

In recent years, there has been a growing focus on non-apoptotic regulated cell death (RCD) as a target for cancer drug development. This includes autophagy, necroptosis, pyroptosis, ferroptosis, and copper death. Developing agents that either induce or inhibit RCD holds the potential to hinder tumor occurrence, proliferation, and metastasis while enhancing sensitivity to immunotherapy [159, 160]. Studies reveal that the interaction between ATG proteins during autophagy can drive phase separation and facilitate the formation of autophagosomes. In cancer, targeted modulation of autophagy-induced phase separation can influence the formation and separation of droplet-shaped bodies during autophagy, offering innovative strategies for cancer treatment. For instance, inducers or inhibitors of Beclin-1 can be employed to regulate autophagy formation, inducing or inhibiting autophagy, exerting a tumor-suppressive effect in various breast cancer types. This presents a novel target for treatment and enhances sensitivity to immunotherapy [161]. Additionally, a specific inhibitor of ferroptosis, icFSP1, can induce ferroptosis inhibitor protein-1 (FSP1) to form phase-separated condensates before inhibiting ferroptosis, thereby promoting tumor cell ferroptosis. This renders icFSP1 a potential target drug for treating refractory solid tumors [66]. However, the molecular mechanisms related to phase separation in other forms of cell death and the potential combined treatment effects between immunotherapies targeting each cell death mechanism remain unclear. The effectiveness of corresponding regulatory drugs in clinical practice also requires optimization. Hence, there is an urgent need to develop effective new modulators in the future. Simultaneously, understanding the mechanisms of action for each cell death related to phase separation will provide a powerful approach for tumor therapy.

### Condensate modification therapy

Numerous drug development companies are dedicated to advancing drug development in the realm of biomolecular condensates, introducing a therapeutic approach known as condensate-modifying therapeutics (c-mods). The fundamental principle underlying this therapy is to prevent or reverse diseases by regulating the physical properties, macromolecular networks, composition, dynamics, and/or function of specific biomolecular condensates. More precisely, c-mods can address condensate lesions, disrupt the normal function of disease-related condensates, or exert their effects by incapacitating or dissociating the target proteins from the condensate. This innovative approach holds promise for preventing or reversing various diseases, including cancer. Notably, several approved drugs may exert c-mod effects to some extent [162].

In conclusion, the exploration of tumor-associated biomolecular condensates driven by phase separation has emerged as a prominent focus in drug development. These condensates present novel avenues for drug discovery, even for targets previously considered "undruggable" [163], offering innovative methods for the treatment of tumors. However, the complexity and dynamic nature of biomolecular condensates pose challenges in drug development. Hence, in order to attain the intended clinical outcomes, there is a need for future investigations into pharmacological outcomes, the composition, structure, and function of specific condensates, as well as their correlation with diseases. These studies are crucial to ensure the effectiveness and safety of interventions.

### Summary and future perspectives

Phase separation, driven by multivalent interactions among biological macromolecules, is intricately regulated by mechanisms like PTMs, membrane surface interactions, molecular chaperones, and RNA. It plays a crucial role in governing diverse physiological functions, including chromosome structure, cell division, signal transduction, gene expression, and cell autophagy. As a molecular mechanism, phase separation is implicated in various processes associated with tumor, influencing carcinogenesis or tumor suppression. Its close ties to tumor occurrence, development, and treatment resistance encompass aspects such as DNA repair, transcriptional regulation, expression of proto-oncogenes and tumor suppressor genes, as well as the formation of PML bodies and SGs. Biomolecular condensates formed through phase separation offer novel targets for molecular intervention in tumor. However, several challenges and considerations persist in this field. Questions abound regarding how to delineate the composition, structure, regulatory mechanisms, and specific physiological functions of these condensates. Additionally, uncertainties linger over whether disrupting the formation of phase-separated biomolecular condensates in one physiological activity might impact other life processes. The exploration of common structures in the phase separation of diverse biological macromolecules, the determination of whether the pathogenic effects of abnormal phase separation in tumors result from the formation of relevant condensates or the loss of physiological functions in biomacromolecules, and the assessment of whether targeted drugs for biomolecular condensates outperform those targeting single molecules are crucial considerations.The future demands urgent attention from researchers to develop new tools, optimize existing technologies, and delve more deeply into the study of phase separation. Resolving current challenges and exploring the condensed matter associated with tumors at a molecular level can offer a new avenue for tumor treatment. This involves adjusting the structure and biological activity of biomolecules condensed matter, providing a promising approach in the fight against tumor.

## Data Availability

Not applicable, please refer to the original references.

## Abbreviations

MBO: Membrane organelles
ER: Endoplasmic reticulum
MLO: Membraneless organelles
SGs: Stress granules
PML: Promyelocytic Leukemia
LLPS: Liquid–liquid phase separation
AD: Alzheimer's disease
IDRs: Intrinsically disordered regions
RNP: Ribonucleoprotein
HP1α: Heterochromatin protein 1α
H3K9me3: Histone H3 lysine 9 methyl
EB1: End-binding protein 1
cGAS: Cyclic GMP-AMP synthase
STING: Stimulator of interferon genes
dsDNA: Double-stranded DNA
cGAMP: Cyclic GMP-AMP
TBK1: TANK-binding enzyme 1
IRF3: Interferon regulatory factor3
PPARγ: Peroxisome proliferator-activated receptor gamma
NR: Nuclear hormone receptor
RXRα: Retinoic acid X receptor alpha
PPRE: Peroxisome proliferation response element
PAS: Pre-autophagosomal structures
PSD: Postsynaptic density
AMPAR: AMPA receptors
SH3: SRC homology 3
PRM: Proline-rich motif
LCRs: Low complexity regions
RBDs: RNA-binding domains
NLRP6: Nucleotide-Binding Oligomerization Domain-Like Receptor Pyrin Domain-Containing Protein 6
dsRNA: Double-stranded RNA
P bodies: Processing Bodies
PTMs: Post-translational modifications
ALS: Amyotrophic lateral sclerosis
FTD: Frontotemporal dementia
E1: Ubiquitin-activating enzymes
E2: Ubiquitin-conjugating enzymes
E3: Ubiquitin-ligase enzymes
PQC: Protein quality control
TNRF6: Tumor necrosis factor receptor (TNFR)-associated factor 6
Sufu: Suppressor of Fused
O-GlcNAcylation: O-acetyl-glucosamine glycosylation
α-syn: α-Synuclein
OGT: O-GlcNAc transferase
OGA: O-GlcNAcase enzyme
TCR: T cell receptor
LAT: T cell activation linker protein
MAPK: Mitogen-activated protein kinase
TNPO1: Transporter protein1
FALS: Familial amyotrophic lateral sclerosis
FTLD: Frontotemporal lobar degeneration
DDX: DEAD-box RNA
LSPT: Liquid–Solid Phase Transition
DDR: DNA damage response
DSBs: DNA double-strand breaks
NHEJ: Non-homologous end joining
HR: Homologous recombination
dilncRNAs: Damage-induced long non-coding RNAs
53BP1: P53-binding protein 1
FOs: Fusion oncoproteins
TFs: Transcription factors
SEs: Super-enhancers
RNAPII: RNA polymerase II
IFN-γ: Interferon-γ
LCDs: Low Complexity Domains
CTD: C-terminal domain
LncRNA: Long non-coding RNA
IGF2BP1: Insulin-like growth factor2 mRNA-binding protein 1
NBs: Nuclear bodies
DAXX: Death domain-associated protein
APL: Acute promyelocytic leukemia
RARα: Retinoic acid receptor α
HDAC6: Histone deacetylase 6
PUFA: Polyunsaturated fatty acids
TZD: Thiazolidinediones
NSCLC: Non-small cell lung cancer
ICB: Immune checkpoint blockade therapy
TME: Tumor microenvironment
PCa: Prostate cancer
AR: Androgen receptor
NTD: N-terminal domain
CRL3: Cullin-Ring E3 ubiquitin ligase
CUL3: Cullin 3
FSP1: Ferroptosis inhibitor protein-1
c-mods: Condensate-modifying therapeutics

## References

[1] Gomes E, Shorter J (2019). The molecular language of membraneless organelles. J Biol Chem.

[2] Ren J, Zhang Z, Zong Z (2022). Emerging implications of phase separation in cancer. Adv Sci (Weinh).

[3] Olins DE, Olins AL (2003). Chromatin history: our view from the bridge. Nat Rev Mol Cell Biol.

[4] Ong JY, Torres JZ. Phase Separation in Cell Division [J]. Mol Cell. 2020;80(1):9–20. 10.1016/j.molcel.2020.08.007 PMC7541545 32860741

[5] Lin CC, Suen KM, Jeffrey PA (2022). Receptor tyrosine kinases regulate signal transduction through a liquid-liquid phase separated state. Mol Cell.

[6] Lafontaine DLJ, Riback JA, Bascetin R (2021). The nucleolus as a multiphase liquid condensate. Nat Rev Mol Cell Biol.

[7] Noda NN, Wang Z, Zhang H. Liquid-liquid phase separation in autophagy [J]. J Cell Biol. 2020;219(8):e202004062. 10.1083/jcb.202004062 PMC7401820 32603410

[8] Boija A, Klein IA, Young RA (2021). Biomolecular Condensates and Cancer. Cancer Cell.

[9] Surewicz WK (2022). Tau liquid-liquid phase separation in neurodegenerative diseases. Trends Cell Biol.

[10] Kenzui T, Nobuyoshi A (2021). Aberrant phase separation and cancer. The FEBS J.

[11] Haneul Y, Catherine T, Allan DD (2019). Cellular sensing by phase separation: Using the process, not just the products. J Biol Chemist.

[12] Alberti S, Gladfelter A, Mittag T (2019). Considerations and challenges in studying liquid-liquid phase separation and biomolecular condensates. Cell.

[13] Boyan S, Zhaoming C, Chunyu Y (2021). Computational Screening of Phase-separating Proteins [J]. Gen Proteomics Bioinform.

[14] Bin W, Lei Z, Tong D (2021). Liquid-liquid phase separation in human health and diseases. Signal Trans Targeted Ther.

[15] Pederson T. The nucleolus. Cold Spring Harb Perspect Biol. 2011;3(3):a000638. 10.1101/cshperspect.a000638 PMC3039934 21106648

[16] Wilson EB (1899). The structure of protoplasm. Science.

[17] Matsui SI, Seon BK, Sandberg AA (1979). Disappearance of a structural chromatin protein A24 in mitosis: implications for molecular basis of chromatin condensation. Proc Natl Acad Sci U S A.

[18] Adlakha RC, Rao PN (1986). Molecular mechanisms of the chromosome condensation and decondensation cycle in mammalian cells. BioEssays.

[19] Brangwynne CP, Eckmann CR, Courson DS (2009). Germline P granules are liquid droplets that localize by controlled dissolution/condensation. Science.

[20] Brangwynne CP, Mitchison TJ, Hyman AA. Active liquid-like behavior of nucleoli determines their size and shape in Xenopus laevis oocytes. Proc Natl Acad Sci U S A. 2011;108(11):4334–9. 10.1073/pnas.1017150108 PMC3060270 21368180

[21] Pilong L, Sudeep B, Hui-Chun C, et al. Phase transitions in the assembly of multivalent signalling proteins [J]. Nature. 2012;483(7389):336–40. 10.1038/nature10879 PMC3343696 22398450

[22] Kato M, Han TW, Xie S (2012). Cell-free formation of RNA granules: low complexity sequence domains form dynamic fibers within hydrogels. Cell.

[23] Lin Y, Protter DSW, Rosen MK (2015). Formation and maturation of phase-separated liquid droplets by RNA-binding proteins. Mol Cell.

[24] Wegmann S, Eftekharzadeh B, Tepper K (2018). Tau protein liquid-liquid phase separation can initiate tau aggregation. Embo J.

[25] Rubio K, Dobersch S, Barreto G (2019). Functional interactions between scaffold proteins, noncoding RNAs, and genome loci induce liquid-liquid phase separation as organizing principle for 3-dimensional nuclear architecture: implications in cancer [J]. Faseb j.

[26] Alberti S, Dormann D (2019). Liquid-liquid phase separation in disease. Annu Rev Genet.

[27] Tian S, Curnutte HA, Trcek T (2020). RNA Granules: A View from the RNA Perspective. Molecules.

[28] Gibson BA, Doolittle LK, Schneider MWG (2019). Organization of chromatin by intrinsic and regulated phase separation. Cell.

[29] Larson AG, Elnatan D, Keenen MM (2017). Liquid droplet formation by HP1α suggests a role for phase separation in heterochromatin. Nature.

[30] Sanulli S, Trnka MJ, Dharmarajan V (2019). HP1 reshapes nucleosome core to promote phase separation of heterochromatin. Nature.

[31] Ahn JH, Davis ES, Daugird TA (2021). Phase separation drives aberrant chromatin looping and cancer development. Nature.

[32] Song X, Yang F, Yang T (2023). Phase separation of EB1 guides microtubule plus-end dynamics. Nat Cell Biol.

[33] Pan J, Fei CJ, Hu Y (2023). Current understanding of the cGAS-STING signaling pathway: Structure, regulatory mechanisms, and related diseases. Zool Res.

[34] Li Y, Peng J, Xia Y (2023). Sufu limits sepsis-induced lung inflammation via regulating phase separation of TRAF6. Theranostics.

[35] Li Z, Luo L, Yu W (2022). PPARγ phase separates with RXRα at PPREs to regulate target gene expression. Cell Discov.

[36] Fujioka Y, Alam JM, Noshiro D (2020). Phase separation organizes the site of autophagosome formation. Nature.

[37] Zheng H, Peng K, Gou X (2023). RNA recruitment switches the fate of protein condensates from autophagic degradation to accumulation. J Cell Biol.

[38] Yang S, Liu C, Guo Y (2022). Self-construction of actin networks through phase separation-induced abLIM1 condensates. Proc Natl Acad Sci U S A.

[39] Chen X, Jia B, Araki Y (2022). Arc weakens synapses by dispersing AMPA receptors from postsynaptic density via modulating PSD phase separation. Cell Res.

[40] Li P, Banjade S, Cheng HC (2012). Phase transitions in the assembly of multivalent signalling proteins. Nature.

[41] Nott TJ, Petsalaki E, Farber P (2015). Phase transition of a disordered nuage protein generates environmentally responsive membraneless organelles. Mol Cell.

[42] Wang J, Choi JM, Holehouse AS (2018). A molecular grammar governing the driving forces for phase separation of prion-like RNA binding proteins. Cell.

[43] Qamar S, Wang G, Randle SJ (2018). FUS Phase Separation Is Modulated by a Molecular Chaperone and Methylation of Arginine Cation-π Interactions. Cell.

[44] Shen C, Li R, Negro R (2021). Phase separation drives RNA virus-induced activation of the NLRP6 inflammasome. Cell.

[45] Gruijs Da Silva LA, Simonetti F, Hutten S (2022). Disease-linked TDP-43 hyperphosphorylation suppresses TDP-43 condensation and aggregation. Embo J.

[46] Sun D, Wu R, Zheng J (2018). Polyubiquitin chain-induced p62 phase separation drives autophagic cargo segregation. Cell Res.

[47] Dao TP, Kolaitis RM, Kim HJ (2018). Ubiquitin modulates liquid-liquid phase separation of UBQLN2 via disruption of multivalent interactions. Mol Cell.

[48] Marotta NP, Lin YH, Lewis YE (2015). O-GlcNAc modification blocks the aggregation and toxicity of the protein α-synuclein associated with Parkinson's disease. Nat Chem.

[49] Jones N, Blasutig IM, Eremina V (2006). Nck adaptor proteins link nephrin to the actin cytoskeleton of kidney podocytes. Nature.

[50] Huang WY, Yan Q, Lin WC (2016). Phosphotyrosine-mediated LAT assembly on membranes drives kinetic bifurcation in recruitment dynamics of the Ras activator SOS. Proc Natl Acad Sci U S A.

[51] Gao Y, Zhu Y, Wang H (2022). Lipid-mediated phase separation of AGO proteins on the ER controls nascent-peptide ubiquitination. Mol Cell.

[52] Zheng Q, Chen Y, Chen D (2022). Calcium transients on the ER surface trigger liquid-liquid phase separation of FIP200 to specify autophagosome initiation sites. Cell.

[53] Panier S, Boulton SJ (2014). Double-strand break repair: 53BP1 comes into focus. Nat Rev Mol Cell Biol.

[54] Alghoul E, Paloni M, Takedachi A (2023). Compartmentalization of the SUMO/RNF4 pathway by SLX4 drives DNA repair. Mol Cell.

[55] Udayakumar D, Dynan WS (2015). Characterization of DNA binding and pairing activities associated with the native SFPQ·NONO DNA repair protein complex. Biochem Biophys Res Commun.

[56] Shirnekhi HK, Chandra B, Kriwacki RW (2023). The role of phase-separated condensates in fusion oncoprotein-driven cancers. Ann Rev Cancer Biol.

[57] Tulpule A, Guan J, Neel DS (2021). Kinase-mediated RAS signaling via membraneless cytoplasmic protein granules. Cell.

[58] Hnisz D, Shrinivas K, Young RA (2017). A phase separation model for transcriptional control. Cell.

[59] Lu Y, Wu T, Gutman O (2020). Phase separation of TAZ compartmentalizes the transcription machinery to promote gene expression. Nat Cell Biol.

[60] Liu Q, Li J, Zhang W (2021). Glycogen accumulation and phase separation drives liver tumor initiation. Cell.

[61] Zhu P, He F, Hou Y (2021). A novel hypoxic long noncoding RNA KB-1980E6.3 maintains breast cancer stem cell stemness via interacting with IGF2BP1 to facilitate c-Myc mRNA stability. Oncogene.

[62] Kanapathipillai M. Treating p53 Mutant Aggregation-Associated Cancer [J]. Cancers (Basel). 2018;10(6):154. 10.3390/cancers10060154 PMC6025594 29789497

[63] Mu ZM, Le XF, Vallian S (1997). Stable overexpression of PML alters regulation of cell cycle progression in HeLa cells. Carcinogenesis.

[64] Wang ZG, Delva L, Gaboli M (1998). Role of PML in cell growth and the retinoic acid pathway. Science.

[65] Zhang F, Biswas M, Massah S (2023). Dynamic phase separation of the androgen receptor and its coactivators key to regulate gene expression. Nucleic Acids Res.

[66] Nakamura T, Hipp C, Santos Dias Mourão A (2023). Phase separation of FSP1 promotes ferroptosis. Nature.

[67] Liu X, Liu X, Wang H (2020). Phase separation drives decision making in cell division. J Biol Chem.

[68] Tiwary AK, Zheng Y (2019). Protein phase separation in mitosis [J]. Curr Opin Cell Biol.

[69] Fang R, Jiang Q, Yu X (2022). Recent advances in the activation and regulation of the cGAS-STING pathway. Adv Immunol.

[70] Christofides A, Konstantinidou E, Jani C (2021). The role of peroxisome proliferator-activated receptors (PPAR) in immune responses. Metabolism.

[71] Janani C, Ranjitha Kumari BD (2015). PPAR gamma gene–a review. Diabetes Metab Syndr.

[72] Direnzo J, Söderstrom M, Kurokawa R (1997). Peroxisome proliferator-activated receptors and retinoic acid receptors differentially control the interactions of retinoid X receptor heterodimers with ligands, coactivators, and corepressors. Mol Cell Biol.

[73] Klionsky DJ, Petroni G, Amaravadi RK (2021). Autophagy in major human diseases. Embo j.

[74] Feng Z, Chen X, Wu X (2019). Formation of biological condensates via phase separation: Characteristics, analytical methods, and physiological implications. J Biol Chem.

[75] Hong K, Song D, Jung Y (2020). Behavior control of membrane-less protein liquid condensates with metal ion-induced phase separation. Nat Commun.

[76] Simon JR, Carroll NJ, Rubinstein M (2017). Programming molecular self-assembly of intrinsically disordered proteins containing sequences of low complexity. Nat Chem.

[77] Lin Y, Currie SL, Rosen MK (2017). Intrinsically disordered sequences enable modulation of protein phase separation through distributed tyrosine motifs. J Biol Chem.

[78] Putnam A, Thomas L, Seydoux G (2023). RNA granules: functional compartments or incidental condensates?. Genes Dev.

[79] Bevilacqua PC, Williams AM, Chou HL (2022). RNA multimerization as an organizing force for liquid-liquid phase separation. RNA.

[80] Weber SC, Brangwynne CP (2015). Inverse size scaling of the nucleolus by a concentration-dependent phase transition. Curr Biol.

[81] Kuznetsova IM, Zaslavsky BY, Breydo L (2015). Beyond the excluded volume effects: mechanistic complexity of the crowded milieu. Molecules.

[82] Ruff KM, Roberts S, Chilkoti A (2018). Advances in understanding stimulus-responsive phase behavior of intrinsically disordered protein polymers. J Mol Biol.

[83] Xuejiao J, Min Z, Shuxin C (2022). Effects of pH alterations on stress- and aging-induced protein phase separation [J]. Cell Mole Life Sci.

[84] Snead WT, Gladfelter AS (2019). The control centers of biomolecular phase separation: how membrane surfaces, PTMs, and active processes regulate condensation. Mol Cell.

[85] Gallego LD, Schneider M, Mittal C (2020). Phase separation directs ubiquitination of gene-body nucleosomes. Nature.

[86] Vaughan RM, Kupai A, Rothbart SB (2021). Chromatin regulation through ubiquitin and ubiquitin-like histone modifications. Trends Biochem Sci.

[87] Yasuda S, Tsuchiya H, Kaiho A (2020). Stress- and ubiquitylation-dependent phase separation of the proteasome. Nature.

[88] Li X, Pinou L, Du Y (2023). Emerging roles of O-glycosylation in regulating protein aggregation, phase separation, and functions. Curr Opin Chem Biol.

[89] Yang X, Qian K (2017). Protein O-GlcNAcylation: emerging mechanisms and functions. Nat Rev Mol Cell Biol.

[90] Banjade S, Rosen MK (2014). Phase transitions of multivalent proteins can promote clustering of membrane receptors. Elife.

[91] Kholodenko BN, Hoek JB, Westerhoff HV (2000). Why cytoplasmic signalling proteins should be recruited to cell membranes. Trends Cell Biol.

[92] Donnelly SK, Weisswange I, Zettl M (2013). WIP provides an essential link between Nck and N-WASP during Arp2/3-dependent actin polymerization. Curr Biol.

[93] Su X, Ditlev JA, Hui E (2016). Phase separation of signaling molecules promotes T cell receptor signal transduction. Science.

[94] Snead WT, Jalihal AP, Gerbich TM (2022). Membrane surfaces regulate assembly of ribonucleoprotein condensates. Nat Cell Biol.

[95] Baumann S, Pohlmann T, Jungbluth M (2012). Kinesin-3 and dynein mediate microtubule-dependent co-transport of mRNPs and endosomes. J Cell Sci.

[96] Liao YC, Fernandopulle MS, Wang G (2019). RNA Granules hitchhike on lysosomes for long-distance transport, using annexin A11 as a molecular tether. Cell.

[97] Hartl FU, Bracher A, Hayer-Hartl M (2011). Molecular chaperones in protein folding and proteostasis. Nature.

[98] Burmann BM, Gerez JA, Matečko-Burmann I (2020). Regulation of α-synuclein by chaperones in mammalian cells. Nature.

[99] Hervás R, Oroz J (2020). Mechanistic Insights into the role of molecular chaperones in protein misfolding diseases: from molecular recognition to amyloid disassembly. Int J Mol Sci.

[100] Cordin O, Banroques J, Tanner NK (2006). The DEAD-box protein family of RNA helicases. Gene.

[101] Cargill M, Venkataraman R, Lee S. DEAD-Box RNA Helicases and Genome Stability [J]. Genes (Basel). 2021;12(10):1471. 10.3390/genes12101471 PMC8535883 34680866

[102] Jain S, Wheeler JR, Walters RW (2016). ATPase-modulated stress granules contain a diverse proteome and substructure. Cell.

[103] Shen H, Yanas A, Owens MC (2022). Sexually dimorphic RNA helicases DDX3X and DDX3Y differentially regulate RNA metabolism through phase separation. Mol Cell.

[104] Henninger JE, Oksuz O, Shrinivas K (2021). RNA-mediated feedback control of transcriptional condensates. Cell.

[105] Banerjee PR, Milin AN, Moosa MM (2017). Reentrant phase transition drives dynamic substructure formation in ribonucleoprotein droplets. Angew Chem Int Ed Engl.

[106] Roden C, Gladfelter AS (2021). RNA contributions to the form and function of biomolecular condensates. Nat Rev Mol Cell Biol.

[107] Liu Z, Zhang S, Gu J (2020). Hsp27 chaperones FUS phase separation under the modulation of stress-induced phosphorylation. Nat Struct Mol Biol.

[108] Rass E, Willaume S, Bertrand P. 53BP1: Keeping It under Control, Even at a Distance from DNA Damage [J]. Genes (Basel). 2022;13(12):2390. 10.3390/genes13122390 PMC9778356 36553657

[109] Espinosa JR, Joseph JA, Sanchez-Burgos I (2020). Liquid network connectivity regulates the stability and composition of biomolecular condensates with many components. Proc Natl Acad Sci U S A.

[110] Pessina F, Giavazzi F, Yin Y (2019). Functional transcription promoters at DNA double-strand breaks mediate RNA-driven phase separation of damage-response factors. Nat Cell Biol.

[111] Kilic S, Lezaja A, Gatti M (2019). Phase separation of 53BP1 determines liquid-like behavior of DNA repair compartments. Embo j.

[112] Galanty Y, Belotserkovskaya R, Coates J (2012). RNF4, a SUMO-targeted ubiquitin E3 ligase, promotes DNA double-strand break repair. Genes Dev.

[113] Fan XJ, Wang YL, Zhao WW (2021). NONO phase separation enhances DNA damage repair by accelerating nuclear EGFR-induced DNA-PK activation. Am J Cancer Res.

[114] Mertens F, Johansson B, Fioretos T (2015). The emerging complexity of gene fusions in cancer. Nat Rev Cancer.

[115] Davis RB, Moosa MM, Banerjee PR (2022). Ectopic biomolecular phase transitions: fusion proteins in cancer pathologies. Trends Cell Biol.

[116] Wang Y, Yu C, Pei G (2023). Dissolution of oncofusion transcription factor condensates for cancer therapy. Nat Chem Biol.

[117] Bradner JE, Hnisz D, Young RA (2017). Transcriptional addiction in cancer. Cell.

[118] Sengupta S, George RE (2017). Super-enhancer-driven transcriptional dependencies in cancer. Trends Cancer.

[119] Tong X, Tang R, Xu J (2022). Liquid-liquid phase separation in tumor biology. Signal Transduct Target Ther.

[120] Cai D, Feliciano D, Dong P (2019). Phase separation of YAP reorganizes genome topology for long-term YAP target gene expression. Nat Cell Biol.

[121] Zhu G, Xie J, Fu Z (2021). Pharmacological inhibition of SRC-1 phase separation suppresses YAP oncogenic transcription activity. Cell Res.

[122] Yu M, Peng Z, Qin M (2021). Interferon-γ induces tumor resistance to anti-PD-1 immunotherapy by promoting YAP phase separation. Mol Cell.

[123] Kwon I, Kato M, Xiang S (2013). Phosphorylation-regulated binding of RNA polymerase II to fibrous polymers of low-complexity domains. Cell.

[124] Ryan JJ, Sprunger ML, Holthaus K (2019). Engineered protein disaggregases mitigate toxicity of aberrant prion-like fusion proteins underlying sarcoma. J Biol Chem.

[125] Soragni A, Janzen DM, Johnson LM (2016). A Designed Inhibitor of p53 aggregation rescues p53 tumor suppression in ovarian carcinomas. Cancer Cell.

[126] Ruggero D, Wang ZG, Pandolfi PP (2000). The puzzling multiple lives of PML and its role in the genesis of cancer. BioEssays.

[127] Escobar-Cabrera E, Okon M, Lau DK (2011). Characterizing the N- and C-terminal Small ubiquitin-like modifier (SUMO)-interacting motifs of the scaffold protein DAXX. J Biol Chem.

[128] Mahmud I, Liao D (2019). DAXX in cancer: phenomena, processes, mechanisms and regulation. Nucleic Acids Res.

[129] Delbarre E, Ivanauskiene K, Spirkoski J (2017). PML protein organizes heterochromatin domains where it regulates histone H3.3 deposition by ATRX/DAXX. Genome Res.

[130] Lallemand-Breitenbach V, De Thé H (2018). PML nuclear bodies: from architecture to function. Curr Opin Cell Biol.

[131] Tang SY, Wan YP, Wu YM (2015). Death domain associated protein (Daxx), a multi-functional protein. Cell Mol Biol Lett.

[132] Tan Y, Wang X, Song H (2021). A PML/RARα direct target atlas redefines transcriptional deregulation in acute promyelocytic leukemia. Blood.

[133] Di Masi A, Cilli D, Berardinelli F (2016). PML nuclear body disruption impairs DNA double-strand break sensing and repair in APL. Cell Death Dis.

[134] Wu W, Tan Y, Yin H (2023). Phase separation is required for PML nuclear body biogenesis and function. Faseb j.

[135] Protter DSW, Parker R (2016). Principles and properties of stress granules. Trends Cell Biol.

[136] Song MS, Grabocka E (2023). Stress Granules in Cancer [J]. Rev Physiol Biochem Pharmacol.

[137] Yang P, Mathieu C, Kolaitis RM (2020). G3BP1 Is a tunable switch that triggers phase separation to assemble stress granules. Cell.

[138] Maurya PK, Mishra A, Yadav BS (2017). Role of Y Box Protein-1 in cancer: as potential biomarker and novel therapeutic target. J Cancer.

[139] Somasekharan SP, El-Naggar A, Leprivier G (2015). YB-1 regulates stress granule formation and tumor progression by translationally activating G3BP1. J Cell Biol.

[140] El-Naggar AM, Somasekharan SP, Wang Y (2019). Class I HDAC inhibitors enhance YB-1 acetylation and oxidative stress to block sarcoma metastasis. EMBO Rep.

[141] Peng Q, Tan S, Xia L (2022). Phase separation in cancer: from the impacts and mechanisms to treatment potentials. Int J Biol Sci.

[142] Augimeri G, Bonofiglio D (2021). PPARgamma: a potential intrinsic and extrinsic molecular target for breast cancer therapy. Biomedicines.

[143] Augimeri G, Giordano C, Gelsomino L (2020). The role of PPARγ ligands in breast cancer: from basic research to clinical studies. Cancers (Basel).

[144] Ansorge N, Dannecker C, Jeschke U (2021). Combined COX-2/PPARγ expression as independent negative prognosticator for vulvar cancer patients. Diagnostics (Basel).

[145] Shao W, Kuhn C, Mayr D (2020). Cytoplasmic PPARγ is a marker of poor prognosis in patients with Cox-1 negative primary breast cancers. J Transl Med.

[146] Kim TW, Hong DW, Park JW (2020). CB11, a novel purine-based PPARɣ ligand, overcomes radio-resistance by regulating ATM signalling and EMT in human non-small-cell lung cancer cells. Br J Cancer.

[147] Xiong Z, Chan SL, Zhou J (2023). Targeting PPAR-gamma counteracts tumour adaptation to immune-checkpoint blockade in hepatocellular carcinoma. Gut.

[148] Xie J, He H, Kong W (2022). Targeting androgen receptor phase separation to overcome antiandrogen resistance. Nat Chem Biol.

[149] Yao H, Xu J (2020). Regulation of cancer immune checkpoint: mono- and poly-ubiquitination: tags for fate. Adv Exp Med Biol.

[150] Li XM, Zhao ZY, Yu X (2023). Exploiting E3 ubiquitin ligases to reeducate the tumor microenvironment for cancer therapy. Exp Hematol Oncol.

[151] Wang P, Song J, Ye D (2020). CRL3s: the BTB-CUL3-RING E3 ubiquitin ligases. Adv Exp Med Biol.

[152] Bouchard JJ, Otero JH, Scott DC (2018). Cancer mutations of the tumor suppressor spop disrupt the formation of active, phase-separated compartments. Mol Cell.

[153] Chen HY, Chen RH (2016). Cullin 3 ubiquitin ligases in cancer biology: functions and therapeutic implications. Front Oncol.

[154] Deng L, Meng T, Chen L (2020). The role of ubiquitination in tumorigenesis and targeted drug discovery. Signal Transduct Target Ther.

[155] Li Q, Gao P. Phase separation in cGAS-STING signaling [J]. Front Med, 2023. 10.1007/s11684-023-1026-6 37906339

[156] Li T, Huang T, Du M (2021). Phosphorylation and chromatin tethering prevent cGAS activation during mitosis. Science.

[157] Volkman HE, Cambier S, Gray EE, et al. Tight nuclear tethering of cGAS is essential for preventing autoreactivity. Elife. 2019.8:e47491. 10.7554/eLife.47491 PMC6927687 31808743

[158] Fang L, Hao Y, Yu H (2023). Methionine restriction promotes cGAS activation and chromatin untethering through demethylation to enhance antitumor immunity. Cancer Cell.

[159] Tong X, Tang R, Xiao M (2022). Targeting cell death pathways for cancer therapy: recent developments in necroptosis, pyroptosis, ferroptosis, and cuproptosis research. J Hematol Oncol.

[160] Gao W, Wang X, Zhou Y (2022). Autophagy, ferroptosis, pyroptosis, and necroptosis in tumor immunotherapy. Signal Transduct Target Ther.

[161] Jung YY, Lee YK, Koo JS (2016). The potential of Beclin 1 as a therapeutic target for the treatment of breast cancer. Expert Opin Ther Targets.

[162] Mitrea DM, Mittasch M, Gomes BF (2022). Modulating biomolecular condensates: a novel approach to drug discovery. Nat Rev Drug Discov.

[163] Biesaga M, Frigolé-Vivas M, Salvatella X (2021). Intrinsically disordered proteins and biomolecular condensates as drug targets. Curr Opin Chem Biol.

